# Influence of Doping on the Topological Surface States of Crystalline Bi_2_Se_3_ Topological Insulators

**DOI:** 10.3390/ma15062083

**Published:** 2022-03-11

**Authors:** Kamil Nowak, Michał Jurczyszyn, Maciej Chrobak, Krzysztof Maćkosz, Andrii Naumov, Natalia Olszowska, Marcin Rosmus, Ireneusz Miotkowski, Andrzej Kozłowski, Marcin Sikora, Marek Przybylski

**Affiliations:** 1 Faculty of Physics and Applied Computer Science, AGH University of Science and Technology, 30-059 Krakow, Poland; knowak@agh.edu.pl (K.N.); mchrobak@agh.edu.pl (M.C.); mackosz@agh.edu.pl (K.M.); kozlow@agh.edu.pl (A.K.); marprzyb@agh.edu.pl (M.P.); 2 Academic Centre for Materials and Nanotechnology, AGH University of Science and Technology, 30-059 Krakow, Poland; naumov@agh.edu.pl (A.N.); marcins@agh.edu.pl (M.S.); 3 National Synchrotron Radiation Centre, Jagiellonian University, 30-392 Krakow, Poland; natalia.olszowska@uj.edu.pl (N.O.); marcin.rosmus@uj.edu.pl (M.R.); 4 Marian Smoluchowski Institute of Physics, Jagiellonian University, 30-348 Krakow, Poland; 5 Department of Physics and Astronomy, Purdue University, West Lafayette, IN 47907, USA; irek@purdue.edu

**Keywords:** topological insulators, Bi_2_Se_3_, magnetic dopants, structural defects, local electronic structure

## Abstract

We present STM/STS, ARPES and magnetotransport studies of the surface topography and electronic structure of pristine Bi_2_Se_3_ in comparison to Bi_1.96_Mg_0.04_Se_3_ and Bi_1.98_Fe_0.02_Se_3_. The topography images reveal a large number of complex, triangle-shaped defects at the surface. The local electronic structure of both the defected and non-defected regions is examined by STS. The defect-related states shift together with the Dirac point observed in the undefected area, suggesting that the local electronic structure at the defects is influenced by doping in the same way as the electronic structure of the undefected surface. Additional information about the electronic structure of the samples is provided by ARPES, which reveals the dependence of the bulk and surface electronic bands on doping, including such parameters as the Fermi wave vector. The subtle changes of the surface electronic structure by doping are verified with magneto-transport measurements at low temperatures (200 mK) allowing the detection of Shubnikov-de Haas (SdH) quantum oscillations.

## 1. Introduction

Topological insulators (TIs) are a relatively new type of materials that are characterized by the simultaneous occurrence of a bulk energy gap and peculiar metallic surface states. These metallic boundaries originate as a consequence of a topologically nontrivial bulk electronic structure resulting from strong spin-orbit coupling. Such surface states form characteristic Dirac cones (DCs) with linear dispersion relation, spin—momentum locking [1] (chiral spin texture) and topological protection from backscattering on non-magnetic impurities. Doping can be used to shift the Fermi level (E_F_) into the bulk bandgap, and thus, to realize the surface quantum topological transport exclusively.

The topological phenomena occur at the interface between insulators with a trivial topology (e.g., vacuum) and those with a non-trivial topology (volumetric TI). Thus, the formation of surface states by closing the bandgap at the interface is an indispensable mechanism of the transition between systems of different topologies.

Three-dimensional topological insulators were experimentally realized first in Bi_1-x_Sb_x_ single crystals [2], with surface bands mapped in an angle–resolved photoemission spectroscopy (ARPES) measurement. Then, the topological insulator behavior was found in Bi_2_Se_3_ and Bi_2_Te_3_ [3,4]. Single crystals of Bi_2_Se_3_ show a bulk bandgap of about 0.3 eV and a single Dirac cone located outside the bulk bands [5]. Such an electronic structure makes Bi_2_Se_3_ a very promising candidate both for research on unique phenomena related to its non-trivial topology and potential applications in modern electronic devices. These include thermoelectric applications [6], optoelectronics [7], next-generation memory technologies [8] and many others. Furthermore, the large energy gap means that topological properties are manifested not only at extremely low temperatures but can be investigated and used even at room temperature. The main complication about these materials is bulk residual conductivity arising from structural defects and impurities, which are often revealed [9].

Soon after the discovery of the topological order in Bi_2_Se_3_, it was realized that the bottom of the conduction band and topological surface states coexist at the Fermi level [4]. This n-type behavior occurs as a consequence of vacancies and native anti-site defects. Taking full advantage of the topological properties of this material requires compensation of this native n-type doping and shifting the Fermi level into the bandgap, where the density of bulk electronic states vanishes. The quantum topological transport would then be realized at the surface only and the density of the carriers would be highly tunable using an applied electrical field. Therefore, high quality p-type crystals are critically important for basic as well as applied research on Bi_2_Se_3_, and a great deal of research work has been devoted to manipulating the Fermi level by searching for suitable p-type dopants. These efforts include volumetric doping with elements, such as Mn [10], Sn [4], Fe [11,12], Ca [13,14], Mg [15], Cr [16,17], Pb [18], surface adsorption with Fe [19,20], Co [21], and controlling a number of native anti-site defects [22,23,24]. Doping with magnetic materials is particularly important due to the fact that the magnetic order leads to the breaking of the time reversal symmetry, and thus, can destroy the topological order. Indeed, magnetic dopants can open the energy gap at the Dirac point of a topological surface state [4]. This effect leads to the creation of odd multiples of Dirac fermions [20] and opens up a new branch related to magnetic topological insulators [25].

Despite the fact that the destruction of the topological electronic states by magnetic dopants of high concentration has been demonstrated [20], the question still open is whether and how a slight deviation from the perfect stoichiometry in Bi_2_Se_3_ affects its DC structure of topological surface states and the electronic structure in general. This includes both the location of the E_F_ as well as the surface and volume states in relation to each other. Another question is the influence of defects on the electronic structure of a TI. Since defects cause a change in the local electronic structure by introducing additional electronic states, the vital question is how the electronic structure around the defects responds to the changes in the host electronic structure caused by the introduction of dopants.

The aim of this paper is to shed some more light on the questions posed above and to determine how: (1) small changes in Bi_2_Se_3_ stoichiometry caused by dopants (of concentrations below 2%) affect the surface topological states, i.e., the characteristic Dirac cone, (2) such small changes in Bi_2_Se_3_ stoichiometry affect the volume electronic structure of a TI and determine the Fermi level, in particular, whether its shift from the conduction band to the energy gap would be possible, and (3) the surface electronic states resulting from structural defects respond to the changes of the volume electronic structure caused by dopants of low concentration (if such changes take place), i.e., do they shift to the energy gap or not?

As mentioned above, doping with magnetic atoms can destroy the topological order. The still open question is whether even amounts of dopants on the level of 1% introduce such changes. Therefore, the main aim of our study is to clarify better how such small concentrations of both magnetic and non-magnetic dopants in Bi_2_Se_3_ affect its nontrivial topology and the topological surface states. To address these questions, a rarely seen in literature combined observations of local topography (STM), electronic structure (STS, ARPES) and magnetoresistance (which allows observing SdH quantum oscillations) were conducted. The results are discussed in comparison to the modified electronic structure as obtained in the recent theoretical calculations [26,27].

## 2. Experimental

The subject of this research are single crystals of pristine, Mg- and Fe-doped bismuth selenide with stoichiometry Bi_2_Se_3_, Bi_1.96_Mg_0.04_Se_3_ and Bi_1.98_Fe_0.02_Se_3_. The samples were synthesized via the Bridgman method. Initially, 5 N purity elements were deoxidized in carbon boats in a two-zone horizontal furnace in a vacuum of 10^−5^ Pa and then purified further by multiple vacuum distillations under a dynamic vacuum of 10^−5^ Pa until they reached 7N purity [28]. The initial synthesis was performed in the same boats at 1170 K, after which the material was slowly cooled under a controlled Se vapor pressure. Then, the initially synthesized material was transferred to a quartz ampoule that, after evacuation, was placed in a 3-zone vertical furnace. The radial temperature gradient in the growth zone was estimated to be less than 0.5 K/cm. The linear gradient in the growth zone was set to 5 K/cm. The ampoule velocity across the growth zone was 0.5 to 1.5 mm/h, resulting in the cooling velocity of 0.25–0.75 K/h. As a result, easily cleavable along the a−b plane and contamination free samples were obtained. The presence of dopants was confirmed by a Secondary Ion Mass Spectroscopy (SIMS) experiment using the TOF-SIMS spectrometer (IONTOF, Münster, Germany) and X-ray absorption spectroscopy (XAS) experiment carried out in the National Centre for Synchrotron Radiation (NCSR) SOLARIS. The crystallographic structure was examined by X-Ray Diffraction (XRD) using the Empyrean diffractometer (Panalytical, Malvern, UK).

Prior to the STM and ARPES measurements, the samples were prepared by cleaving in-situ in an ultra-high vacuum (UHV) with the base pressure below 2 × 10^−7^ Pa at room temperature, which resulted in obtaining an atomically clean (0001) surface.

The crystallographic quality of the samples was tested using low-energy electron diffraction (LEED). The measurements were performed at room temperature using the retarding field analyzer (SPECTALEED, Omicron, Taunusstein, Germany).

The topography and surface electronic states were probed with a variable-temperature scanning tunneling microscope (VT STM) with an Beetle scan head (RHK, Troy, MI, USA) (controlled by an R9plus SPM control system, RHK, Troy, MI, USA). The STM and STS measurements were carried in UHV under a base pressure of 10^−8^ Pa at room temperature. The differential tunneling conductance (dI/dV) was taken via the standard lock-in technique with a 20 mV modulation amplitude and 12.3 kHz frequency. An electrochemically etched tungsten tip was used. The fast Fourier transform (FFT) power spectra of the raw STM topographies were calculated using the WSXM software (5.0 DEVELOP, Madrid, Spain) [29]. The STM/STS investigations were carried out directly after cleaving.

The ARPES measurements were performed at the UARPES beamline of NCSR SOLARIS at pressures better than 1 × 10^−8^ Pa using horizontally polarized undulator radiation. Photoelectrons were detected with a DA30L electron energy analyzer (Scienta Omicron, Uppsala, Sweden). The overall resolutions of the ARPES measurements were 8 meV (energy) and 0.1 deg (angular). The samples were glued with epoxy resin to a sample holder and cleaved in UHV. The measurements were carried out as soon as possible after cleaving and cooling down to 12 K, having in mind the aging effect of the sample surface.

The magnetic field dependence of the longitudinal and transverse resistivity was measured on samples cut to a cuboid shape with a base of 5 × 5 mm and a height around 0.2 mm. Each sample, before being put into a refrigerator, was cleaved at ambient conditions using scotch tape, and then gold wires were connected to the corners with silver paste. Thus, the point contacts were prepared to measure electrical conductivity by using the AC Van der Pauw method. This method was chosen instead of the classic Hall bar because it was easier to prepare all the samples of the same shape and size. However, Hall bar geometry was tested as well to be sure that both methods give the same results. The electrical transport measurements were performed at a temperature of 200 mK in a TRITON dilution refrigerator (Oxford Instruments, Abingdon, UK) using Nanonis Tramea measurement system (SPECS, Berlin, Germany) with a lock-in amplifier. A magnetic field of up to 14 T was applied at varying angles with respect to the sample surface.

## 3. Results

### 3.1. Scanning Tunneling Microscopy

Scanning tunneling microscopy (STM) is a powerful tool for investigating surface topography and, in the spectroscopy mode (STS), the surface electronic structure with a probing region was confined to a single defect or dopant.

**Figure 1:**
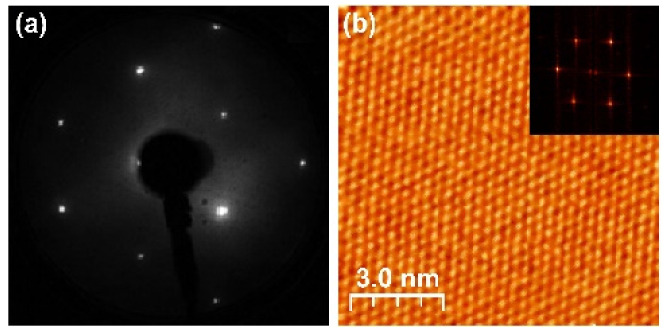
The surface of the Bi_2_Se_3_ sample at room temperature. (**a**) LEED pattern at 61 eV, (**b**) 10 × 10 nm STM topography image acquired at V_bias_ = −430 mV, I = 200 pA. The FFT power spectrum (inset) calculated from the raw STM topography image in (**b**).

The surface crystal structure of the investigated samples was characterized using the LEED and high resolution STM topography imaging methods. The results for the pristine Bi_2_Se_3_ and doped Bi_1.96_Mg_0.04_Se_3_ and Bi_1.98_Fe_0.02_Se_3_ crystals are very similar, which is rational considering that the samples contain 1–2 at% of dopants. In Figure 1 we present representative results for the UHV cleaved Bi_2_Se_3_ sample. In Figure 1a, we show the LEED pattern recorded with a primary beam energy of 61 eV. The pattern shows six well-pronounced single atomic-lattice Bragg spots, which can be grouped into a hexagon. This result, collected from the global area, is confirmed by the 10 × 10 nm atomically resolved STM topography image shown in Figure 1b. The FFT power spectrum presented in the inset is calculated from an STM topography scan (Figure 1b). The hexagonal symmetry of the surface lattice is clearly visible in both local (by STM) and global (by LEED) measurements.

**Figure 2:**
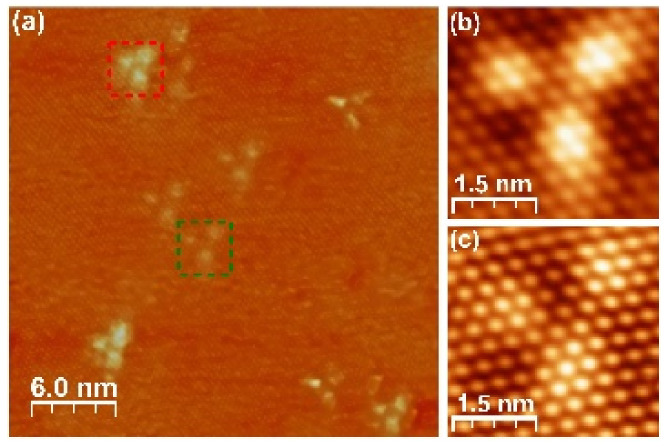
STM topography scans recorded at room temperature, V_bias_ = −340 mV, I = 300 pA. (**a**) 30 × 30 nm STM image of the Bi_2_Se_3_ surface, revealing the most frequent types of defects in the 5th and 6th atomic layer marked with the red and green square, respectively. (**b**,**c**) high resolution STM topography scans depicting surface images of the defect in the 5th atomic layer (**b**) and in the 6th atomic layer (**c**) in Bi_2_Se_3_.

The topography images of all the investigated samples reveal a large number of triangle-shaped defects at the surface (Figure 2). Some of the previous works based on a combination of STM research and Density Functional Theory (DFT) simulation state that the defects exhibiting the same pattern of surface charge distribution are recognized as Bi-Se antisites [23,30]. However, as the dominant type of defects, the authors of [23] found Se vacancies. Such a statement cannot be verified by comparison with other experimental data, since the formation of a particular type of defect results from certain conditions under which the single crystals were grown. In our selection of scanning parameters, a vast majority of the defects we observe correspond to the substitutions of Bi for Se in the fifth and sixth atomic layers as identified based on the calculations and observations reported in [23,30], while the Se vacancies are invisible. In Figure 2a, we present a typical 30 × 30 nm STM topography scan revealing these two types of defects marked with red and green, respectively. High resolution scans of the specified defects are presented in Figure 2b,c for the Bi for Se substitution defects in the fifth and sixth atomic layer, respectively.

**Figure 3:**
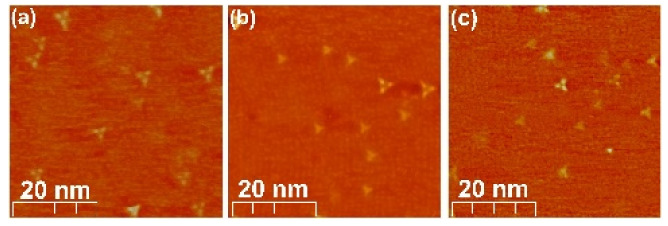
50 × 50 nm STM topography scans of (**a**) Bi_2_Se_3_, (**b**) Bi_1.96_Mg_0.04_Se_3_, (**c**) Bi_1.98_Fe_0.02_Se_3_, revealing the density of the defects on the studied surfaces (obtained at V_bias_ = −340 mV and I = 300 pA).

We used STM imaging to investigate the samples in terms of the density and type of defects. Figure 3 presents the comparison of the sample topographies at given tunneling parameters. The defects, widely investigated and reported with pristine Bi_2_Se_3_ [23,30] (Figure 3a), are also found on the surfaces of the Mg-doped (Figure 3b) and Fe-doped (Figure 3c) samples. The STM topography images reveal randomly distributed defects, the density of which is very similar for all the samples studied. Most of the visible defects occur as a consequence of the substitutions of Bi in the Se positions in the 5th and 6th atomic layer [23] and are not associated with the presence of a specific dopant. However, there are other features in the STM images that are visible after more careful observation. They are also of a triangular shape, but smaller than the features identified as substitutions of Bi for Se in the 5th and 6th atomic layers. More interestingly, they exist only for the doped samples and are not visible for the pristine Bi_2_Se_3_. Their number is larger for the sample doped with Mg compared to the sample doped with Fe, in accordance with the concentration of Mg and Fe dopants, respectively.

### 3.2. Scanning Tunneling Spectroscopy

**Figure 4:**
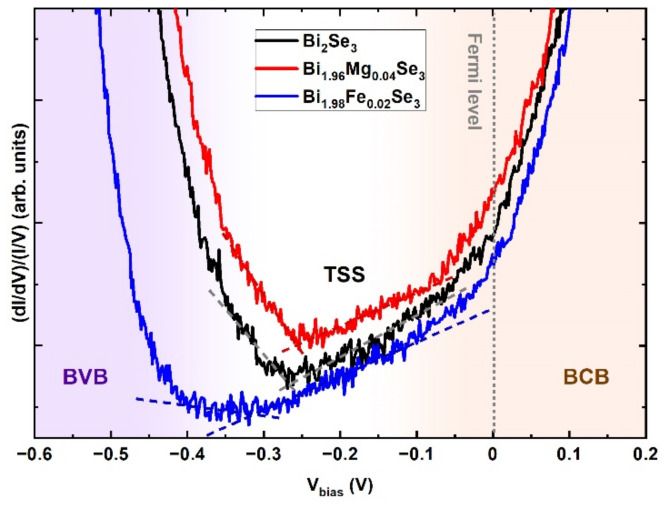
Normalized STS spectra measured at room temperature at positions between the defects, i.e., on structurally perfect areas of the Bi_2_Se_3_, Bi_1.96_Mg_0.04_Se_3_ and Bi_1.98_Fe_0.02_Se_3_ surfaces. The regions of the BVB, BCB and TSS are marked with the violet, orange and white background, respectively. The segments with a linear dispersion relation are marked with lines.

After identifying the origin of the observed defects (Section 3.1), we compared the surface electronic structure of the pristine Bi_2_Se_3_ sample to that of the samples doped with Fe and Mg. The electronic structure was examined by STS, which is a measure of the local density of states (LDOS) as a function of the bias voltage. At first, in order to avoid the influence of the defects on the LDOS, only points outside the defect area were selected for the measurements. Since the tunneling current depends both on the states of the surface being tested and on the states of the tip [31], the dI/dV spectra were normalized by dividing the differential conductance dI/dV by the total conductance I/V, i.e., (dI/dV)/(I/V) [32]. The divergence problem at small bias voltages in the case of (dI/dV)/(I/V) was overcome by broadening ΔV up to 1.2 V [33]. All the spectra were acquired at a tunneling current of 150 pA. The results for the points outside the defect area, obtained at room temperature, are presented in Figure 4, where the averaged and normalized STS spectra of the investigated samples are compared.

In all the measured spectra presented in Figure 4, it is possible to observe the upper edge of the bulk valence band (BVB) and the lower edge of the bulk conduction band (BCB). The finite density of states inside the bulk energy gap is due to the topological surface states (TSSs). The spectrum of the pristine Bi_2_Se_3_ is n-type, as expected [3]. The spectra of the doped samples are also n-type, with a similar Fermi level located in the BCB. However, depending on dopants we observe some differences in the BCB and TSS regions. The doping with Fe results in a slight shifting of the edge of the BCB towards low energies and a broadening of the range of the linearly dispersed surface states. The doping with Mg has almost no effect: the range of the linearly dispersed states seems to be unchanged with respect to the pristine Bi_2_Se_3_.

**Figure 5:**
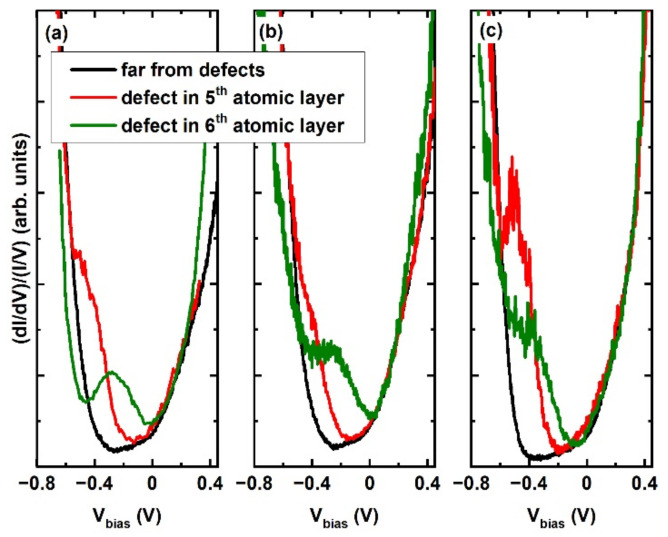
Normalized STS spectra measured at room temperature in positions far from the defects (black), at the 5th atomic layer defect (red) and at the 6th atomic layer defect (green) on (**a**) Bi_2_Se_3_, (**b**) Bi_1.96_Mg_0.04_Se_3_, (**c**) Bi_1.98_Fe_0.02_Se_3_, respectively.

As mentioned in the previous paragraph, the electronic structure of the surface has changed due to doping, at least in the case of the Fe-doped sample. As the surface topography images of both the pristine and doped crystals reveal the same structural Bi for Se substitutions defects, the vital question is how the electronic states of the defects interact with this modified electronic structure of the doped samples. In Figure 5, we present the STS results obtained both in the area of the 5th (red) and 6th (green) atomic layer defects and between the defects (black). Figure 5a presents the results for the pristine Bi_2_Se_3_. Both the investigated defects introduce additional electronic states visible as local maxima. The positions of the local maxima are obtained by fitting two additional Lorentz-shape peaks to the STS spectra. The results of such fits are summarized in Table 1.

It is clear that the positions of the peaks qualitatively coincide with the position of the Dirac point of the undisturbed surface structure. This result confirms previous simulations [30], and early STS results [34]. The states with a linear dispersion relation are not visible in the area of the defects due to the dominating defect-related electronic states. Figure 5b presents the results for Bi_1.96_Mg_0.04_Se_3_, for which the defected areas reveal similar defect-induced states. The positions of the corresponding maxima are not remarkably shifted with respect to the pristine sample. The opposite situation occurs in the case of Bi_1.98_Fe_0.02_Se_3_ (Figure 5c). The defect-induced electronic states are shifted away from the Fermi level to lower energies. The defect-related states seem to reflect the electronic structure of the undefected surface: if the positions of the maxima related to the defect-induced states do shift, they do it facilely by changing the DP position.

### 3.3. Angle–Resolved Photoemission Spectroscopy

**Figure 6:**
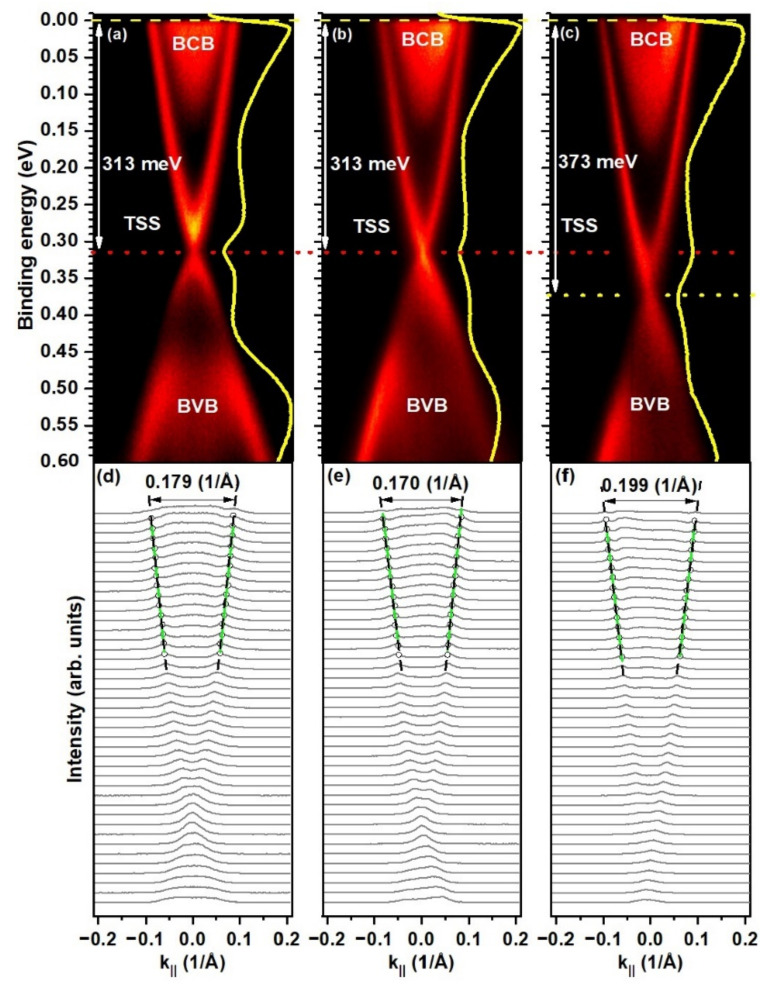
The ARPES results obtained at a photon energy of 17.5 eV for the Bi_2_Se_3_ sample (**a**), Bi_1.96_Mg_0.04_Se_3_ (**b**), and Bi_1.98_Fe_0.02_Se_3_ (**c**). The relative positions of the Fermi level and the Dirac point are labeled. The yellow lines represent the integrals of the ARPES intensity along the k_||_ (in-plane momentum) direction. Corresponding momentum distribution curves for Bi_2_Se_3_ (**d**), Bi_1.96_Mg_0.04_Se_3_ (**e**), and Bi_1.98_Fe_0.02_Se_3_ (**f**) obtained from (**a**–**c**), respectively. The profiles were collected every 10 meV from the E_F_ to 400 meV. The TSSs are marked with the lines extrapolated to the E_F_, which allowed to determine the diameters of the Fermi surfaces.

The ARPES experiment was performed to find the relation between the local electronic structure probed by STS mostly from the near-surface atomic layers and the electronic structure probed by the electrons emerging from much deeper atomic layers (and from a larger area). In Figure 6, we present the ARPES intensity spectra measured in one direction in reciprocal space: for Bi_2_Se_3_ (Figure 6a), Bi_1.96_Mg_0.04_Se_3_ (Figure 6b), and Bi_1.98_Fe_0.02_Se_3_ (Figure 6c). All the spectra reveal a BCB, TSS in the form of a Dirac cone, and a BVB. We observe that the Mg dopants do not influence remarkably the position of the edges of the bulk electronic bands with respect to the pristine Bi_2_Se_3_. For pristine and Mg-doped samples, the conduction band ends at an energy of about 140 meV below the E_F_. The upper edge of the valence band appears at an energy of about −460 meV. In the case of the Fe-doped sample, the bottom edge of BCB appears at 170 meV below the E_F_, and the upper edge of BVB appears at about −490 meV. The surface states with a linear dispersion relation are not gapped and show the DP at 313 meV below the E_F_ in the case of the Bi_2_Se_3_ (Figure 6a and Table 2) and Bi_1.96_Mg_0.04_Se_3_ (Figure 6b). The DP is shifted to −373 meV for Bi_1.98_Fe_0.02_Se_3_ (Figure 6c).

Figure 6d–f presents the momentum distribution curves obtained from the ARPES spectra shown in Figure 6a–c, respectively. The TSSs are marked with lines drawn through the centers of the fitted Lorentz functions. The line was extrapolated to the Fermi level. The intersections mark the edge of the Fermi surface, which allows determining the diameter of the Fermi surfaces as 2∙k_F_ (where k_F_ is the Fermi wave vector; assuming a circular cross section of the Fermi surface in the k_||_ plane), which equals to 0.180 Å^−1^ for the non-doped material (Figure 6d). This diameter is shortened to 0.174 Å^−1^ by the introduction of Mg dopants (Figure 6e) and remarkably lengthened to 0.201 Å^−1^ by doping with Fe (Table 2). Such a significant increase of k_F_ in the case of the Fe-doped sample might be related to deviation from the regular circular cross section of the Fermi surface due to E_F_ remarkably shifting with respect to that of the pristine sample. The analysis was carried out for three photon energies: 17.5, 22.5 and 25 eV; the average values are listed in Table 2.

The Fermi velocity (v_F_) in the vicinity of the E_F_ was calculated from the linear part of the dispersion relation using the formula:

(1) $$E\left(k\right)=\hbar \cdot v_{F}\cdot k=>{\;v}_{F}=\frac{1}{\hbar }\left(\frac{\mathrm{dE}}{\mathrm{dk}}\right).$$

Finally, knowing k_F_ and v_F_, the effective mass of the charge carriers could be calculated:

(2) $$m^{*}=\frac{\hbar k_{F}}{v_{F}}.$$

The mean values of both the Fermi velocities and effective masses of the carriers are listed in Table 2.

Both in the case of v_F_ and m*, there is again some systematic effect, i.e., the sample doped with Mg shows a larger v_F_ than the pristine sample, whereas the sample doped with Fe shows a smaller v_F_. The tendency is the opposite for m*, which is smaller for the Mg-doped and larger for the Fe-doped samples.

The magnetic doping of Fe does not lead to the opening of a gap in the topological surface states; thus, the Dirac cone is preserved in the case of the Bi_1.98_Fe_0.02_Se_3_ sample (Figure 6c,f).

### 3.4. Magneto-Transport

Finally, the details of the local electronic structure, in particular those concerning the DC formed by the surface electronic states, were investigated by means of magneto-transport measurements at very low temperatures allowing to detect Shubnikov-de Haas (SdH) quantum oscillations (which confirm a high quality of our samples).

**Figure 7:**
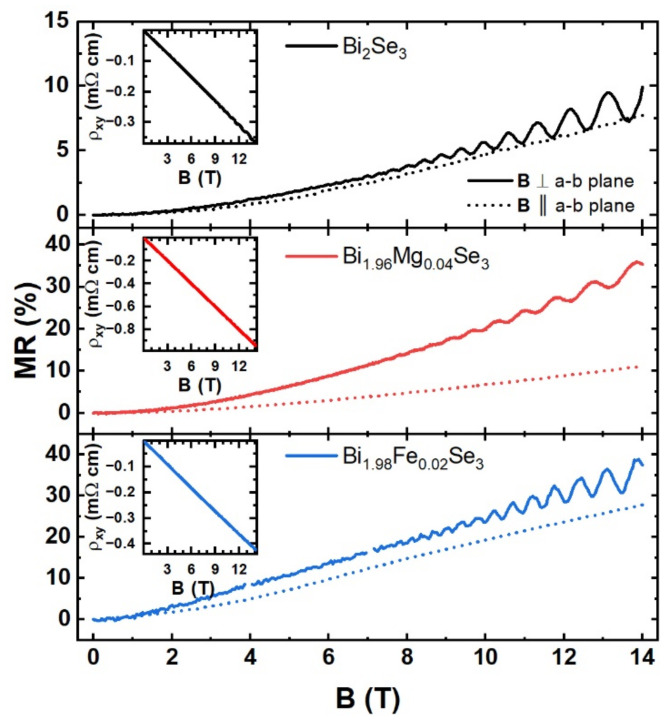
The magnetoresistance of Bi_2_Se_3_, Bi_1.96_Mg_0.04_Se_3_ and Bi_1.98_Fe_0.02_Se_3_ at 200 mK. The solid lines are for B perpendicular to the a-b plane and the dashed lines are for B parallel to the a-b plane. The insets show the transverse resistivity (ρ_xy_).

The results, i.e., the longitudinal MR (MR = 100%∙(ρ_xx_ (B) − ρ_xx_ (0))/ρ_xx_ (0)) and transverse (ρ_xy_) resistivity probed at 200 mK versus an external magnetic field for the direction of the magnetic field perpendicular and parallel to the a-b plane are shown in Figure 7. The negative Hall resistivity proves that the charge carriers are electrons (the insets in Figure 7) for all the samples. From the low field magnetic dependence of the transverse resistivity, the carrier concentrations and their mobility were obtained and listed in Table 3.

The SdH oscillations are easily discerned from ρ_xx_ as well as the QHE plateau of ρ_xy_, which proves that the Landau levels cross the Fermi surface with an increasing magnetic field. Since ρ_xy_ ≈ ρ_xx_, the oscillations should be extracted from σ_xx_ instead from ρ_xx_, otherwise, the phase of the oscillations may be incorrectly determined. The σ_xx_ conductivity is given by the formula [35]:

(3) $$\sigma_{\mathrm{xx}}=\frac{\rho_{\mathrm{xx}}}{\rho_{\mathrm{xx}}^{2}{+\rho }_{\mathrm{xy}}^{2}}.$$

**Figure 8:**
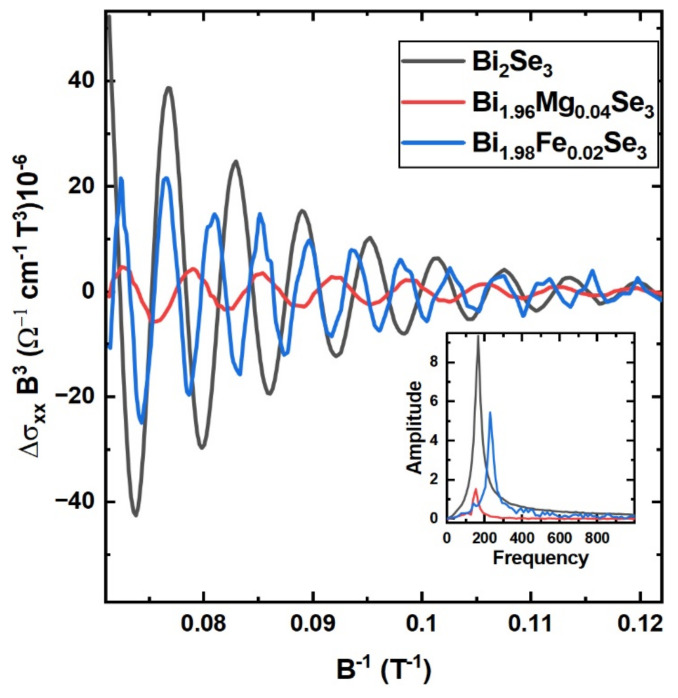
The Shubnikov-de Haas oscillations plotted as Δσ_xx_B^3^ vs. the inverse magnetic field of Bi_2_Se_3_, Bi_1.96_Mg_0.04_Se_3_ and Bi_1.98_Fe_0.02_Se_3_ at 200 mK. The inset shows the spectral intensity of the SdH oscillations.

To obtain pure SdH oscillations from the field dependence of σ_xx_, the smooth background fitted using the asymmetric least square method was extracted. The resulting Δσ_xx_, plotted as Δσ_xx_B^3^ vs. 1/B, is shown in Figure 8. Multiplication of Δσ_xx_ by B^3^ is intended to visualize better the oscillations above the noise level. This procedure does not affect the frequency of SdH oscillations. Single frequency oscillations were observed for each sample, which is confirmed by the FFT analysis shown in the inset of Figure 8. The frequency and amplitude of the oscillations depend on doping (more precisely, the amplitude decreases for both dopants, whereas the frequency decreases for Mg and increases for the Fe doped sample).

## 4. Discussion

This work was focused on the experimental investigation of the electronic structure of pristine, Mg- and Fe-doped bismuth selenide. Despite the concentration of 1–2 at% of dopants in our samples, their presence had already been confirmed by other techniques (SIMS, XAS). No additional peaks in the XRD measurements were observed (not shown here), which testifies the lack of a separated phase due to the aggregating dopants.

The global crystallographic surface structure characterized by LEED and the local topographic features measured by STM indicates that the surface structure of all studied samples is similar. Here, we used the STM technique to compare the tested samples from the defects point of view, using a suitable set of tunneling parameters.

The STM experiment allowed us to confirm that all the measured samples, regardless of the presence of dopants, are characterized by a similar density of structural defects in the form of Bi substitutions for Se positions. These defects are clearly visible in chosen tunneling parameters and clearly indicated as the substitutions of Bi in the Se positions in the 5th and 6th atomic layers (i.e., at the end of the first and at the beginning of the next QL). The identification was performed by comparison with literature data [23,30] having in mind both how the individual defects change the surface topography and the position of maxima in corresponding STS measurements. From the literature, the native n-type character of the studied material results from the existence of donor-like defects (e.g., Se vacancies). On the other hand, the substitutional Bi defects at the Se sites are an amphoteric dopant that plays an acceptor role for strong n-type Bi_2_Se_3_ [34,36,37].

However, there are also triangular features visible in the STM images in the number proportional to the concentration of the dopants and not observed for the pristine Bi_2_Se_3_ sample. They can originate from the dopants placed closer to the surfaces, and thus appear to be smaller than the substitutions of Bi in the Se positions in the 5th and 6th atomic layers. The shape of the features is similar for both Mg and Fe-doped samples, which is not surprising since the features do not reflect the dopants but the defects caused by the dopants. Nevertheless, we cannot indicate them as characteristic features that can be unambiguously associated with the presence of the dopants in the given places, since no theoretical modeling is available. The limited visibility of such defects could be related to the chosen tunneling parameters, which can hinder a clear observation of the dopants related perturbation in the topographic images (in particular in the case when the dopant concentration is small). Moreover, such defects might not be the only way in which the dopants manifest their presence, and thus their number is smaller than one could expect.

We start our discussion with a clean surface, free of defects. STS was used to identify the local density of states. The finite density of states inside the bulk energy gap is due to topological surface states. The characteristic structures of the surface states in the form of a DC are preserved despite the presence of dopants. This means that dopants introduced at a concentration of 1–2 at% do not remarkably influence the topological surface states of the studied materials. In other words, since the DC is the result of nontrivial topology, neither nonmagnetic Mg nor magnetic Fe dopants destroy this topology, but subtle changes to the electronic structure are introduced.

The absolute position of the edges of the BCB and BVB changes, while the distance between them (energy gap) remains unchanged. The ARPES spectra (Figure 6) show a bulk bandgap of about 320 meV for all three investigated samples.

The Fermi level is located in the BCB both for the pristine and doped samples. The results allow stating that the Fermi level is not affected by doping at a low concentration of Mg (in the Mg-doped Bi_2_Se_3_ sample the DP is placed at the energy 313 meV below E_F_, which is exactly the position of the DP in the undoped sample). On the other hand, the E_F_ is clearly shifted towards higher energies, i.e., deeper into the BCB as a result of doping by low concentration of Fe (in the case of doping with Fe, the position of DP is changed to 373 meV with respect to E_F_). Changing the TSS may only appear as a consequence of changing the structure of the bulk bands, which is visible from Figure 6 (in particular for the Fe-doped sample). Thus, our experiment clearly confirms the results of the DFT calculations of the bulk electronic structure of the Fe-doped Be_2_Se_3_ published recently [26,27].

Unfortunately, in STS measurements is not easy to quantify the energy range of the linearly dispersed surface states because the electrons tunneling from the surface states overlap with the electrons tunneling from the valence band. Nevertheless, qualitatively there is a nice agreement of the STS spectra with the results from the ARPES experiments, both showing the same tendency: shifting of DP towards low energies for Fe- and no change for Mg-doped sample with respect to the pristine Bi_2_Se_3_.

The electronic structure of the studied samples differs with the diameter of the Fermi surface as well, which can be related to the presence of dopants and quantitatively is related to the carrier density. The changes in k_F_ show it is slightly smaller for the Mg-doped and noticeably larger for the Fe-doped samples with respect to the pristine Bi_2_Se_3_ sample.

The shift of the DP vs. the E_F_ and changing k_F_ by doping coincide with changes in the dispersion relation of the surface states near the E_F_, which are detectable both by STS and ARPES. From the linear dispersion relation in the vicinity of E_F_, both the Fermi velocity and effective mass can be calculated.

The characteristic structures of the surface states in the form of a DC are preserved despite the presence of dopants as confirmed by analyzing the quantum oscillations in the magnetotransport measurements (see Section 3.4). For all the measured samples, a single oscillation frequency can be extracted from the FFT spectra (the inset in Figure 8). The frequency of SdH oscillations (f_SdH_) is directly related to the cross section (A_F_) of the extremal Fermi surface in momentum space following the Onsager relation [38]:

(4) $$f_{\mathrm{SdH}}=\left(\frac{h}{4\pi^{2}e}\right)A_{F},$$

where $A_{F}=\pi k_{F}^{2}$, k_F_ is the Fermi wave vector, e is the electron charge and h is the Planck constant. The Fermi wave vector is linked to the surface carrier density [39] by 𝑛_2__𝐷_ = $k_{F}^{2}$/4𝜋. In our case, for the pristine sample, we obtain the frequency of 165 T (see Table 3), in good agreement with data reported in the literature [40,41,42,43]. The Fermi wave vector for this frequency is 0.071 Å^−1^, so the carrier concentration is 3.98 × 10^12^ cm^−2^. For the Mg-doped sample, the oscillations frequency is 152 T, which results in the 0.068 Å^−1^ Fermi wave vector and the concentration equal to 3.67 × 10^12^ cm^−2^. Fe doping results in the opposite effect of Mg doping for f_SdH_. The obtained frequency is 230 T, which leads to the 0.084 Å^−1^ Fermi wave vector and the carrier concentration of 5.55 × 10^12^ cm^−2^.

Based on the analysis of the quantum oscillations, we can say that doping affects the Fermi wave vector, which is smaller for the Mg-doped and larger for the Fe-doped sample with respect to the undoped Bi_2_Se_3_ sample. These systematics coincide qualitatively with the Fermi wave vector obtained from the ARPES analysis, which results in k_F_ = 0.090 Å^−1^, 0.087 Å^−1^ and 0.100 Å^−1^ (Section 3.3, Table 3), respectively. Such non-perfect quantitative agreement is not surprising, having in mind that the SdH oscillations were detected at 200 mK, whereas the ARPES experiment was carried out at 12 K. In the ARPES experiments, the band bending of the electronic bands near to the surface is observed, and thus influences the values of k_F_. Based on the analysis of the quantum oscillations, we can say that doping affects the carrier concentration, i.e., for Mg, it decreases and for Fe, it increases with respect to the pristine sample.

Another issue to consider is the origin of the oscillations, i.e., whether they come from the bulk or surface states. In the case of the SdH oscillations originating from surface states (2D), their amplitude should decay with the decreasing component of the external magnetic field perpendicular to the sample surface. This is exactly what is measured for our samples: the oscillations are not visible when a magnetic field is applied parallel to the sample surface (Figure 7) [44]. It is worth mentioning that the Fermi surface is not an ideal sphere (warping effect), which can result in the amplitude of the SdH oscillations being dependent on the orientation of the magnetic field, although this cannot cause the oscillations to disappear. In addition, one can compare the carrier concentrations obtained from the frequency of the SdH oscillations with those measured by the Hall effect. If the SdH oscillations result from the bulk states, the period of the SdH oscillations must be related to a 3D Fermi surface. If we consider the case where the Fermi surface is an ideal sphere, then the carrier concentrations for the obtained Fermi wave vectors, calculated from 𝑛_3__𝐷_ = $\frac{k_{F}^{3}}{3\pi^{2}}$ (assuming states degeneracy with respect to the spin), will be 1.20 × 10^19^ cm^−3^, 1.06 × 10^19^ cm^−3^ and 1.97 × 10^19^ cm^−3^, respectively, for the pristine, Mg- and Fe-doped sample, i.e., following the tendency observed for the shift of the DP vs. the Fermi level and for the k_F_. These results are inconsistent with the Hall effect measurements (Table 3). It should be mentioned, however, that such a comparison cannot be considered sufficient, since the SdH oscillations at high magnetic fields are preferentially sensitive to the carriers of higher mobility whereas the Hall measurements are sensitive to all carriers including even those of low mobility in the bulk band.

Defects in small concentrations do not change the global structure of the topological surface states of the studied materials, which is concluded from the tunneling probing far from the defects and is discussed above. However, both kinds of defects (the 5th and the 6^th^ atomic layer) introduce additional electronic states visible as local maxima, which obstruct the visibility of the surface states with the linear dispersion relation in the area of the defects. The positions/energies of the maxima related to the defect-induced states shift facilely by changing the DP position of the undisturbed surface structure. In other words, the local electronic structure at the defects is influenced by the E_F_ position. The defect-induced states do not destroy the global TSS, which is in accord with the topological protection of the surface states.

## 5. Conclusions

The STM results show that a relatively small amount of volumetric dopants does not introduce significant changes either to the global surface crystallographic order characterized by LEED or the topography of the Bi_2_Se_3_ surface at room temperature in an STM measurement. The characteristic structures of the surface states in the form of DCs are preserved despite the presence of dopants. Since the DC is the result of nontrivial topology, the amount of dopants on the level of 1–2 at% does not destroy it but introduces changes to the electronic structure. The absolute position of the edges of the BCB and BVB changes (for Fe-doped sample) or not (for Mg-doped sample), while the distance between them (energy gap) of about 320 meV remains unchanged. The Fermi level is rather insensitive to the presence of Mg-dopants. However, in the presence of Fe-dopants E_F_ is slightly shifted with respect to the Dirac point (and bulk electronic bands) towards higher energies, as is shown from ARPES and STS experiments in agreement with recent theoretical calculations. The defect-related states shift together with the DP, suggesting that the local electronic structure at the defects is influenced by doping in the same way as the electronic structure of the undefected surface, i.e., the topological surface states are not affected by defects. Doping affects the Fermi wave vector, which is smaller for the Mg-doped and larger for the Fe-doped sample with respect to the undoped Bi_2_Se_3_ sample. This result is confirmed by the quantum oscillations in the magnetotransport measurements. The Fermi wave vector obtained from the frequency of the SdH oscillations depends on doping showing some systematic effect: it is smaller for the Mg-doped and larger for the Fe-doped sample with respect to the pristine sample. This coincides perfectly with the Fermi wave vectors obtained from the ARPES analysis. Since the carrier densities calculated from the Fermi wave vectors are inconsistent with the Hall effect measurements and the SdH oscillations vanish while the magnetic field is applied parallel to the sample surface, one can conclude that the origin of the SdH oscillations is the surface states. The Fermi level is placed in the bulk conduction band both for the pristine and doped samples despite the fact that it would be profitable to shift it to the bandgap by doping. Finally, we state that the combined observations of the local topography (STM), electronic structure (STS, ARPES) and magnetoresistance (which allows observing SdH quantum oscillations) allowed us to collect complementary results and conclude the subtle changes of the electronic structure by doping on the level of 1%, however, with nontrivial topology and the topological surface states not destroyed.

## Figures and Tables

**Table 1:** The positions of the maxima of the additional electronic states were observed at specific defects using the STS method.

	Bi_2_Se_3_ (V)	Bi_1.96_Mg_0.04_Se_3_ (V)	Bi_1.98_Fe_0.02_Se_3_ (V)
Defect in the 5th atomic layer	−0.41	−039	−0.45
Defect in the 6th atomic layer	−0.26	−0.26	−0.32
DP far from defects (from Figure 4)	−0.27	−0.26	−0.32

**Table 2:** The mean values of the diameters of the Fermi surfaces (2∙k_F_), the Fermi velocities (v_F_) and effective masses (m*) averaged over the values obtained for different excitation energies, for the undoped, doped with Mg and doped with Fe samples obtained from the ARPES experiment at 12 K.

Sample	2∙k_F_ (Å^−1^)	v_F_∙10^5^ (m/s)	m* (m_e_)	DP Position (eV)
Bi_2_Se_3_	0.180	6.788	0.154	−0.313
Bi_1.96_Mg_0.04_Se_3_	0.174	6.968	0.144	−0.313
Bi_1.98_Fe_0.02_Se_3_	0.201	5.940	0.197	−0.373

**Table 3:** The estimated parameters from the magnetoresistance, Hall effect and SdH oscillations obtained at 200 mK.

Sample	ρ_xx_ (B = 0) (mΩ∙cm)	n_Hall_ (10^17^ cm^−3^)	μ (cm^2^/Vs)	SdH Freq (T)	k_F_ (Å^−1^)	n_2D_ (10^12^ cm^−2^)
Bi_2_Se_3_	0.290	2.49	8720	165	0.071	3.98
Bi_1.96_Mg_0.04_Se_3_	0.203	0.95	3245	152	0.068	3.67
Bi_1.98_Fe_0.02_Se_3_	0.059	2.05	5128	230	0.084	5.55

## Data Availability

Not applicable.
